# Autistic Children Quickly Orient Away from Both Eyes and Mouths During Face Observation

**DOI:** 10.1007/s10803-021-05378-x

**Published:** 2022-02-09

**Authors:** Lilja Kristín Jónsdóttir, Janina Neufeld, Terje Falck-Ytter, Johan Lundin Kleberg

**Affiliations:** 1 Uppsala Child and Baby Lab, Department of Psychology, Uppsala University, Box 1225, 751 42 Uppsala, Sweden; 2 Center of Neurodevelopmental Disorders at Karolinska Institutet (KIND), Centre for Psychiatry Research, Department of Women’s & Children’s Health, Karolinska Institutet & Region Stockholm, Stockholm, Sweden; 3 Development and Neurodiversity Lab, Department of Psychology, Uppsala University, Uppsala, Sweden; 4 Swedish Collegium for Advanced Study (SCAS), Uppsala, Sweden; 5 Center for Psychiatry Research, Department of Clinical Neuroscience, Karolinska Institutet & Region Stockholm, Stockholm, Sweden; 6 Department of Molecular Medicine and Surgery, Karolinska Institutet, Stockholm, Sweden

**Keywords:** Autism Spectrum Disorder, Eye gaze, Social attention, Visual scanning

## Abstract

Studies have supported two different hypotheses of reduced eye gaze in people with ASD; gaze *avoidance* and gaze *indifference*, while less is known about the role of anxiety*.* We tested these hypotheses using an eye-tracking paradigm that cued the eyes or mouth of emotional faces. Autistic children (*n* = 12, mean age 7 years) looked faster away from both eyes and mouths than controls (*n* = 22). This effect was not explained by anxiety symptoms. No difference was found in latency towards either area. These results indicate that attentional avoidance of autistic children is not specific to eyes, and that they do not show attentional indifference to eyes compared to controls. Atypicalities in visual scanning in ASD are possibly unrelated to specific facial areas.

**Supplementary Information:**

The online version contains supplementary material available at 10.1007/s10803-021-05378-x.

One of the most prominent deficits of social functioning observed in Autism Spectrum Disorder (ASD) is atypical eye gaze behavior (American Psychiatric Association, 2013; Kliemann et al., 2010; Kleberg, Thorup, et al., 2017; Klin et al., 2002; Tanaka & Sung, 2016). The cognitive mechanisms underlying this impairment are still debated, with two opposing theoretical explanations being most supported and salient in the discourse. These are the *avoidance* hypothesis and the *indifference* hypothesis. Each of these hypotheses is grounded in different theories of cognition in ASD.

The eye gaze *avoidance* hypothesis states that diminished eye gaze in ASD stems from active avoidance of eyes, driven by discomfort or aversive hyper-arousal (Tanaka & Sung, 2016). In support of this hypothesis, studies have reported heightened arousal levels in school age children with ASD (Kylliäinen & Hietanen, 2006) and increased amygdala activation in adults with ASD (Dalton et al., 2005) in response to direct eye gaze, compared to their neurotypical peers. Eye gaze may be particularly diminished in response to threatening emotions, further supporting the avoidance theory (Wang et al., 2018).

The eye gaze *indifference* hypothesis states that autistic people do not perceive others’ eyes as engaging or adaptively informative to the same degree as typically developing individuals (Moriuchi et al., 2017). Such indifference might be a product of a general lack of social motivation in ASD (Chevallier et al., 2012) or by an impaired ability to infer mental states from information displayed in the eye region (Baron-Cohen et al., 1997). In support of this hypothesis, toddlers with ASD have been found to be less likely to orient to eyes spontaneously (Moriuchi et al., 2017), and a preference for non-social distractors (in competition with faces) has been found to be correlated with ASD severity in diagnosed toddlers (Kwon et al., 2019).

A currently unanswered question is the extent to which comorbid anxiety can explain reduced attention to eyes in ASD. Recent studies have suggested that 34–50% of individuals with ASD fulfill the criteria for an anxiety disorder, with higher numbers in adults and high-functioning individuals (Maddox & White, 2015; Simonoff et al., 2008; van Steensel et al., 2011). Anxiety disorders are associated with avoidant visual scanning of faces and eyes with direct gaze, likely reflecting that these stimuli are perceived as aversive (Schmidtendorf et al., 2018; Waters et al., 2014; Weeks et al., 2013). It is therefore possible that gaze avoidance in ASD is explained by anxiety rather than by core ASD symptomatology (Amaral et al., 2003; Kleberg, Högström, et al., 2017; Kleberg, Thorup, et al., 2017), whereas core ASD symptoms may be linked to gaze indifference.

Inconsistent results from previous studies may also relate to differences in age. Studies of toddlers and very young children have mostly supported the indifference hypothesis (Kwon et al., 2019; Moriuchi et al., 2017), while the avoidance hypothesis has support from studies of older children, adolescents and adults (Dalton et al., 2005; Kliemann et al., 2010, 2012; Kylliäinen & Hietanen, 2006). This indicates a possible developmental aspect; an initial lack of neurobiological reward in response to other’s eyes in infancy may develop into avoidance with increasing maturity and social pressures at later stages of development (Fletcher-Watson & Happé, 2019).

To sum up, previous studies have given some support to both the avoidance and the indifference hypotheses, but the literature is inconclusive regarding which theory is best supported. Discrepancies in the previous literature may be explained at least partly by comorbid anxiety and differences in the age groups studied. These inconsistencies may be resolved by validation of experimental paradigms which (1) can be used across age groups and (2) provide the means to study links between eye gaze and symptoms of ASD and anxiety across a range of severity.

The paradigm used in the current study is adapted from Kliemann et al.’s (2010 and 2012) studies of autistic adults. This paradigm makes it possible to quantify and study the presence of avoidance and indifference simultaneously, which provides evidence of the presence of either or both these behaviors at a specific age. In their studies using a similar paradigm, Kliemann et al., (2010, 2012), reported that adults with ASD were more likely to make quick gaze shifts away from the eye region than typically developing controls, supporting the avoidance hypothesis. The current study is, to our knowledge, the first to use this kind of paradigm in a group of young children. This is also, to our knowledge, the first study to examine the association between dimensional anxiety symptoms and orienting from human eyes in this age group.

The first aim of the current study was to test the gaze avoidance and gaze indifference hypotheses by comparing a group of young school age children with ASD and a group of typically developing peers, thereby contributing to a more complete picture of the effects of age on gaze behavior in ASD. Facial stimuli were presented, and participants were cued to first attend to either the eyes or the mouth. As there is some evidence for the effects of specifically threatening emotions on eye gaze (e.g. Wang et al., 2018) we varied the emotional expressions displayed by the faces to ensure generalizability of the effect of initially cueing eyes and mouths. We reasoned that the avoidance hypothesis would predict quicker orienting away from eyes in children with ASD as compared to controls, whereas the indifference hypothesis would predict slower orienting from the mouth to the eyes in children with ASD compared to controls (as measured by latency to first fixation). The second aim was to study the association between ASD and anxiety symptoms and orienting away from human eyes, using a dimensional approach. Based on previous results by Kleberg, Högström, et al. (2017), Kleberg, Thorup, et al. (2017), we reasoned that higher levels of anxiety would predict faster orienting away from eyes, while ASD symptom severity would predict slower orienting towards eyes.

## Methods

### Participants

A total of 38 children, (14 diagnosed with ASD and a control group of 24 typically developing children) participated in the study. The ASD group was recruited through adverts posted in a local school for children with special needs, a local habilitation center, as well as relevant social media groups and in general social media posts. The adverts were directed to parents of children from 6 to 10 years old, either typically developing or with a diagnosis of autism or Asperger syndrome. Parents of children with an ASD diagnosis that had participated in previous studies at Uppsala Child- and Babylab and KIND (Center of Neurodevelopmental Disorders at Karolinska Institutet, Stockholm) were contacted personally. Inclusion criteria for the ASD group were; confirmed diagnosis of ASD or Asperger syndrome, parent-reported ability to sit relatively still and perform a task, and absence of known uncorrected vision- or hearing problems. The typically developing control group was recruited from Uppsala Child- and Babylab’s registry of families who had previously expressed interest in participating in research, as well as from the adverts mentioned above. Inclusion criteria for the control group were; parent-reported absence of any diagnosed mental- or neurodevelopmental disorder, parent-reported ability to sit relatively still and perform a task, and absence of known uncorrected vision- or hearing problems. Informed consent was obtained from the legal guardians of all individual participants included in the study. Data were missing from two participants (one in the ASD group and one in the control group) due to excessive head movement and unwillingness to finish the experiment. Consent was withdrawn for one participant from the control group after testing, and no confirmation of diagnosis was provided for one child in the ASD group. Data from 34 children (12 with ASD and 22 controls) were therefore analyzed. The groups were matched for age, but the proportion of male participants was higher in the ASD group, and children with ASD had fewer valid trials in the analyses related to latency to orient to AOIs. Groups did not differ in the proportion of invalid gaze samples (see Table 1).

**Table 1:** Sample characteristics, number of valid trials for each group of participants and p values of differences

	ASD	Control	*p*
N	12	22	
Mean age in years (standard deviation, SD)	7.7 (1.7)	7.6 (0.8)	0.840
Range in years	5–10	6–9	
*n* girls (boys)	3 (9)	12 (10)	
Mean SCAS-P score (SD)	19.1 (9.5)	14.4 (8.0)	0.139
Range	6–37	5–34	
Mean AQ-Child score (SD)	83.3 (19.5)	40.0 (13.0)	** < 0.001**
Range	43–117	19–66	
IQ proxy (SD)	71.8 (18.1)	107.5 (19.6)	** < 0.001**
Range	40–100	70–155	
WISC IV Vocabulary subtest score (SD)	6.9 (2.9)	11.3 (2.4)	** < 0.001**
WISC IV Matrix Reasoning subtest score (SD)	7.4 (1.4)	10.2 (2.6)	**0.001**
Mean number of valid trials away from eyes, out of 32^a^ (SD)	21.58 (7.0)	29.45 (3.6)	**0.002**
Mean number of valid trials away from mouth, out of 16 (SD)	10.33 (4.3)	14.73 (2.1)	**0.005**
Mean number of valid trials to eyes, out of 16 (SD)	10.92 (3.7)	12.55 (3.6)	0.224
Mean number of valid trials to mouth, out of 32 (SD)	13.58 (6.8)	15.64 (6.5)	0.391
% Invalid samples (SD)	17.16 (15.58)	13.63 (12.71)	0.479

^a^In the experiment, trials cueing fixation to the eyes were twice as many as trials cueing fixation to the mouth (directly to right or left eye and between the eyes), which explains the higher number of valid trials in the “Away from eyes” and “To mouth” conditions.

Values in bold indicate a significant difference between the groups at the 0.05 level

Diagnostic status of the ASD group was confirmed through medical records. Participants in the control group had no psychiatric disorder according to parental report and scored below the diagnostic cut-off of the Autism Quotient: Children’s version (no participant in the control group scored higher than 66 points) (Auyeung et al., 2008). IQ was estimated as the mean of the Vocabulary and Matrix Reasoning subtests from the Swedish version of the Wechsler Intelligence Scale for Children (WISC-IV; Wechsler et al., 2003). Valid results were obtained for 20 children in the control group and 11 children in the ASD group. As can be seen in Table 1, IQ was higher in the control group than the ASD group. The sample size was somewhat larger than in previous studies using a highly similar experimental paradigm, meaning that the study had enough power to detect large effect sizes, as reported in these studies (Kliemann et al., 2010, 2012).

### Questionnaires

Symptoms of anxiety and autistic traits were measured using parent-report scales. The Spence Children’s Anxiety Scale for Parents (SCAS-P; Nauta et al., 2004) was used to assess symptoms of anxiety. SCAS-P scores were obtained from 33 participants (12 with ASD, 21 typically developing). Both groups had mean scores within one standard deviation of the mean of a European validation study (Essau et al., 2002), indicating no significant anxiety symptoms. Symptoms of autism were measured using the Autism Spectrum Quotient-Children’s Version (AQ-Child; Auyeung et al., 2008). AQ-Child scores were obtained from all 34 participants. Autistic traits were higher in the ASD group, as expected, while the groups did not differ on anxiety symptoms (see Table 1).

### Experimental Task

**Fig. 1:**
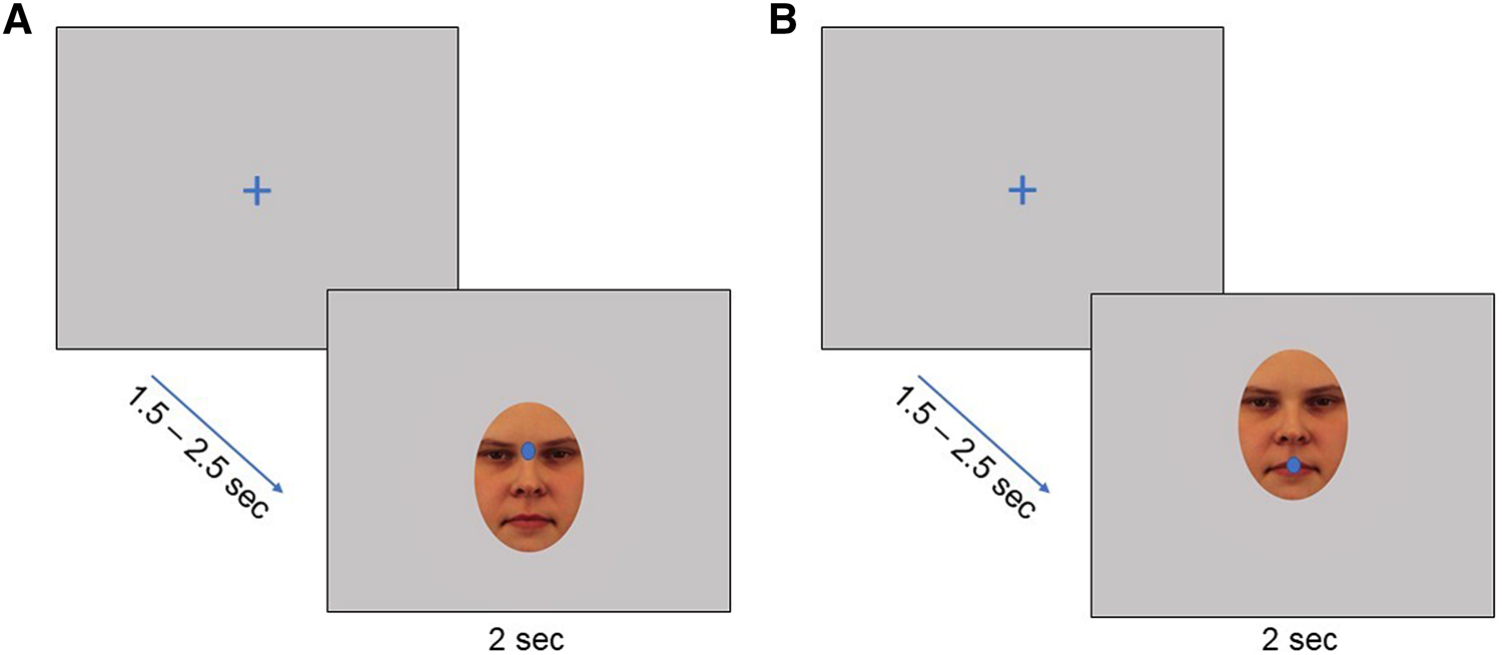
Two examples illustrating single trials within the experimental paradigm. Example **a** shows cueing of initial fixation to the eyes on a female face with a neutral expression. Example **b** shows cueing of initial fixation to the mouth of the same face. Blue dots indicate expected location of initial fixation on the stimulus

A modified version of the experimental task used by Kliemann and colleagues (2010, 2012) was used. The paradigm is illustrated in Fig. 1. Each trial began with a fixation cross placed in the middle of a computer screen, directly followed by a face from the Karolinska Directed Emotional Faces set (Lundqvist et al., 1998), elliptically masked and in full color. Faces displaying expressions depicting anger, happiness, fear and neutrality were randomly presented to ensure generalizability of the results across emotional expressions. The faces were positioned on the screen so that the initial fixation would be either in the eye region or in the mouth region. In total, 48 trials were presented to each participant (32 with initial fixation at the eye area). This difference in number of trials cueing eyes and mouths was due to an exploratory manipulation, in which the initial fixation position within the eye condition was varied to be directed at the left eye, the right eye, or between the eyes. A third of the trials were directed at either eye, a third between the eyes, and a third at the mouth. Preliminary analyses showed that this manipulation did not affect the latency to orient away from an eye AOI created by merging the three eye areas cued in the experiment (*p* > 0.25). The fixation cross was presented for 1.5 s, 1.7 s or 2.5 s, and faces were subsequently presented for 2 s. Duration of fixation cross presentation was varied to prevent potential learning effects. Stimuli were presented on a 17″ computer screen. Facial stimuli displayed a happy, fearful, angry, or neutral expression (equally balanced between stimuli and conditions).

Participants were instructed to maintain gaze at the fixation cross until a face appeared. They were informed that different faces would appear between fixation crosses, and that they were free to view them any way they liked, but to return to the fixation cross when it appeared again.

### Data Recording and Analysis

Gaze data were recorded with a Tobii T120 eye-tracker (Tobii inc, Danderyd, Sweden) with a sampling rate of 60 Hz. Fixations were identified in the raw data using a dispersion-based fixation filter (Tobii fixation filter) with dispersion and duration threshold set to 35 pixels. Gaze shifts away from cued AOIs that occurred earlier than 100 ms after stimulus onset were considered anticipatory and were therefore not analyzed (see Table 2 for each group's number of valid trials in the analyzed conditions). AOIs were defined around the eyes and mouth of the stimuli (both covering 8.33° horizontally and 3.89° vertically). Latency to look away was defined as the time from face stimulus onset to first fixation outside the initially cued AOI (eyes or mouth). Latency to look to an area of interest was defined as the time from face stimuli presentation to first fixation within the relevant area of interest, after disengaging from an initially cued AOI (eyes or mouth).

### Statistical Analysis

Data were analyzed using linear mixed effects regression models. For group comparisons, models with outcome variables “Latency from AOI” and “Latency to AOI” were run, each with fixed factors “Group” (ASD or Control), “Emotion” (Angry, Happy, Fearful or Neutral) and “Region” (initial fixation at Eyes or Mouth). Models included random intercepts for participant (i.e. accounting for repeated measures) and random slope for trial (i.e. accounting for trial order). No results were changed when age was added as covariate. A group difference was found in IQ, and this variable was therefore not added as a covariate due to the known statistical problems associated with controlling for variables which differ between groups of interest (e.g. Miller & Chapman, 2001). In exploratory analyses, main effects of sex and IQ, and interaction terms between sex and group, IQ and group, (in group comparisons) and sex and AQ, and IQ and AQ (in dimensional analyses) were added to the models.

For the dimensional analysis of effects of anxiety and autistic traits on gaze behavior, fixed factors were “AQ scores”, “SCAS-P scores” and “Region”, with outcome variables “Latency from AOI” and “Latency to AOI” averaged at the level of the individual. Statistical significance was tested by χ^2^-tests comparing the model to a null model without the effect of interest (Baayen et al., 2008). Effect sizes for pairwise group comparisons are reported as Cohen’s d, and as Cohen’s f^2^ in the dimensional analyses.

## Results

Results are shown in Tables 2 and 3 and described here.

**Table 2:** Group comparisons (effects from linear mixed effects model)

	χ^2^	*p*	b	SE	d
Latency to orient from AOI					
Group	8.58	**0.003**	205.25	65.69	0.39
Region	52.92	** < 0.001**	− 200.24	27.07	0.42
Emotion	8.52	**0.036**			
Region × group	0.47	0.493	− 41.18	59.98	
Region × emotion	11.07	**0.011**			
Region × emotion × group	0.03	> 0.50			
Latency to orient toward AOI					
Group	0.78	0.376	36.71	40.75	0.06
Region	173.15	** < 0.001**	− 389.90	27.78	0.86
Emotion	0.42	> 0.50			
Region × group	0.95	0.331	− 57.73	59.08	
Region × emotion	4.02	0.260			
Region × emotion × group	0.60	> 0.50			

*AOI* area of interest, *b* unstandardized beta coefficient, *χ*^2^ model comparison chi-square, *d* Cohen’s d.

Values in bold indicate a significant effect at the 0.05 level

**Table 3:** Relation between AQ scores, SCAS-P scores and age, and the latency to orient to, and away from facial regions (effects from linear mixed effects model)

	χ2	*p*	b	SE	*f*^2^
Latency from AOI					
AQ	4.53	**0.033**	− 3.25	1.48	0.03
SCAS-P	1.01	0.314	4.48	4.40	0.01
Age	0.08	0.782	8.36	30.39	< .01
AQ × region	0.08	0.776	− 0.52	1.81	< .01
SCAS-P × region	2.86	0.091	− 9.03	5.15	0.01
Latency to AOI					
AQ	0.04	0.834	− 0.19	0.92	< 0.01
SCAS-P	0.84	0.359	2.54	2.76	< 0.01
Age	0.07	0.784	− 5.19	18.96	< 0.01
AQ × region	0.04	0.850	0.42	2.20	< 0.01
SCAS-P × region	0.23	0.627	− 3.19	6.58	< 0.01

*AOI* area of interest, *AQ* Autism Quotient, *SCAS-P* Spence Children’s Anxiety Scale (parent version), *Region*  mouth or eyes, *b* unstandardized beta coefficient, *χ*^2^ model comparison chi-square, *f*^*2*^ Cohen’s *f*^*2*^

Values in bold indicate a significant effect at the 0.05 level

### Latency to Look Away

**Fig. 2:**
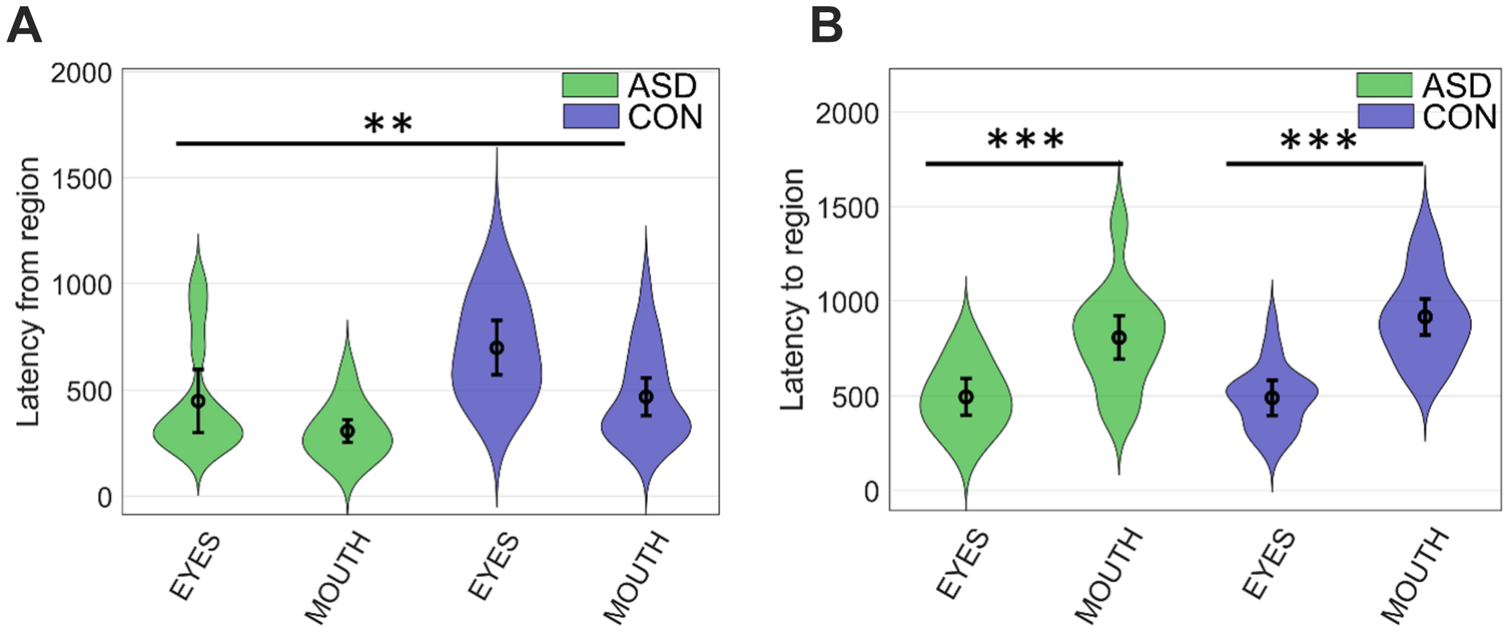
Mean latency to orient away from the initially cued region (**A**), and to the non-cued region (**B**), for the ASD group (shown in green) and control group (shown in blue). Error bars indicate 95% confidence intervals around the mean. Violins show the probability distributions. **p < 0.01 (main effect of group, **A**); ***p < 0.001 (main effect of AOI, **B**)

Children with ASD were quicker to orient away from both the eyes and mouth than TD children (main effect of group, *p* = 0.003) (Fig. 2). There was also a main effect of region, indicating that both groups oriented quicker from the mouth than from the eyes (*p* < 0.001). No significant two- or three-way interactions between group and region or emotion were found (all *p* > 0.30). As can be seen in Table 2, a significant main effect of emotion, and a significant interaction effect between region and emotion were found on latency to look away. Follow-up tests (see Supplementary materials) indicated that the difference in latency to orient from the eyes and mouth was significant for all emotions displayed by the faces, except happiness. Participants were also quicker to look away from any cued region of happy as compared to neutral faces. In an exploratory analysis, main effects of sex and IQ, and interaction effects between sex and group, and group and IQ were added to the model. This did not change any of the results, and no effects involving sex or IQ were significant (all *p* > 0.20).

### Latency to Orient to Eyes and Mouth

The only significant effect was a main effect of region (*p* < 0.001), driven by quicker orienting to eyes than mouths in both groups (Fig. 2).

### Dimensional Analyses

Results are shown in Table 3 and summarized here. Across groups, higher AQ scores predicted quicker orienting away (*p* = 0.029), independent of SCAS-P scores, an effect that was not explained by an AQ × Region interaction (*p* > 0.50). No main or interaction effects involving SCAS-P were found (all *p* > 0.13). These results did not change when main and interaction effects involving sex and IQ were included in the models. All effects involving sex and IQ were statistically non-significant (all *p* > 0.10).

## Discussion

The results of this study indicate that children diagnosed with ASD *orient away from* eyes faster than children with no diagnosis of ASD, but are not slower to *orient to* eyes. These results, by themselves, would lend support to the eye gaze avoidance hypothesis, while not supporting the eye gaze indifference hypothesis. Unexpectedly, however, children with ASD were also quicker than typically developing children to orient away from mouths. This finding is not consistent with the eye gaze avoidance hypothesis, which predicts quicker orienting from eyes specifically, as their highly emotionally expressive nature should render them socially threatening to autistic people (Tanaka & Sung, 2016). It should be noted that the results were not modulated by the emotional expression of the facial stimuli. Taken together, these results might reflect a more general deviation in visual attention allocation in ASD, presenting here as shorter fixations on any cued stimulus. These results are consistent with previous research showing that school age children with ASD display shorter fixations in general during scene perception (Keehn et al., 2008), a phenomenon which may emerge already in infancy (Wass et al., 2015). Although increased scanning may enhance perception in children with ASD in certain ways, as indicated by enhanced performance on the Embedded Figures task (Keehn et al., 2008), it may also be a contributor to the social challenges accompanying the disorder.

Given previous findings of reduced gaze preference for social stimuli in children with ASD (e.g. Kwon et al., 2019; Shaffer et al., 2016), an interesting topic for future research would be to examine how autistic children in this age group attend to specific regions of the face, or the entire face, in the presence of non-social distractors.

As noted in the introduction, gaze avoidance has most consistently been reported in adults with ASD, and rarely in toddlers. In light of our results and previous research, it is possible that a developmental shift towards gaze avoidance takes place during adolescence or late childhood. This is an interesting topic for future studies.

A dimensional analysis supported the findings from the group comparisons, and demonstrated that the link between ASD symptoms and quicker orienting away from facial regions was independent of parent rated anxiety. This suggests that the pattern of quicker reorienting related to ASD symptomatology was not attributable to co-occurring anxiety symptoms. It should, however, be noted that neither group can be described as clinically anxious, based on the obtained SCAS-P scores [no participant scored over 1.25 standard deviations from the mean of a European validation study (Essau et al., 2002)]. An interesting future direction would be to examine the effects of high levels of anxiety on avoidance of and indifference to eye gaze in ASD. A recent study (Kleberg et al., 2021) using a similar task found that adolescents with social anxiety were slower than healthy controls to orient from eyes, but not from mouths.

Our results suggest that an experimental paradigm used previously in adult studies (Kliemann et al., 2010, 2012) is feasible for researching atypical social attention in young children with ASD, but also points to the need to incorporate nonsocial control stimuli in future research. Due to the small sample size, the results must be considered preliminary. The inability to match the groups on intelligence and sex is a limitation for this study. Matching groups on these variables is often a challenge in autism research (Chita-Tegmark, 2016). Low IQ-scores are common in ASD (O’Brien & Pearson, 2004) and such outcomes can be difficult to separate from ASD core symptoms without losing data from a large number of participants. There is redeeming evidence for a non-significant effect of matching of verbal and non-verbal IQ on effect sizes in eye tracking studies of social attention in ASD (Chita-Tegmark, 2016). Future studies should ideally include an IQ matched control group. With regards to sex differences, a growing body of evidence indicates that females with ASD (or female infants at risk) show a more normative pattern of social attention than males with ASD, presenting as a larger preference for social stimuli (Chawarska et al., 2016; Harrop et al., 2018, 2019). As the current study’s experimental group had more boys than girls, sex differences may have skewed the results toward a larger difference between the children with ASD and typically developing children. We did not find evidence for sex differences in the current study, but the small sample size precludes definite conclusions.

Despite these limitations, the results contribute to our understanding of atypical social attention in ASD.

## Supplementary Information

Below is the link to the electronic supplementary material.

Supplementary file1 (PDF 153 kb)
