# Supplementary material for: Autistic Children Quickly Orient Away from Both Eyes and Mouths During Face Observation
Source: J Autism Dev Disord. 2022 Feb 9;53(1):495–502. doi: 10.1007/s10803-021-05378-x (PMC9889486; doi:10.1007/s10803-021-05378-x)
Supplement: Supplementary file 1 — Supplementary file1 (PDF 153 kb) [file 10803_2021_5378_MOESM1_ESM.pdf]

Supplementary Materials

Effects of emotion on the latency to look away. A main effect of emotion was found, $\chi^2 = 8.52$, $p = .036$. Pairwise follow-up comparisons were conducted with a Bonferroni-adjusted alpha level of .008 (correcting for six comparisons). As can be seen in *Table S1*, the only significant effect was a longer latency to look away from the cued region when the face showed a happy as compared to a neutral expression.

Table S1. Pairwise follow-up comparisons of the effects of facial stimuli emotion on latency to look away.

**Effect**	χ^2	**p**	**b**	**SE**	**d**
Angry > Fearful	0.92	.3380	32.30	33.69	0.06
Angry > Happy	2.97	.0850	58.59	33.96	0.12
Neutral > Angry	1.10	.2939	39.95	38.03	0.07
Fearful > Happy	0.90	.3427	30.05	31.66	0.07
Neutral > Happy	7.22	**.0072***	95.57	35.46	0.19
Neutral > Fearful	3.66	.0556	69.05	36.02	0.12

Interaction effect between emotion and AOI on the latency to look away. Effects of AOI (latency from mouth – latency from eyes) for the different emotions are shown in *Figure S1*. Error bars cover estimated marginal means covered by 95% confidence intervals. As can be seen, participants were quicker to orient from the mouth than from the eyes for all emotions except happiness, resulting in an interaction effect between emotion and AOI ($\chi^2 = 11.07$, p = .011).

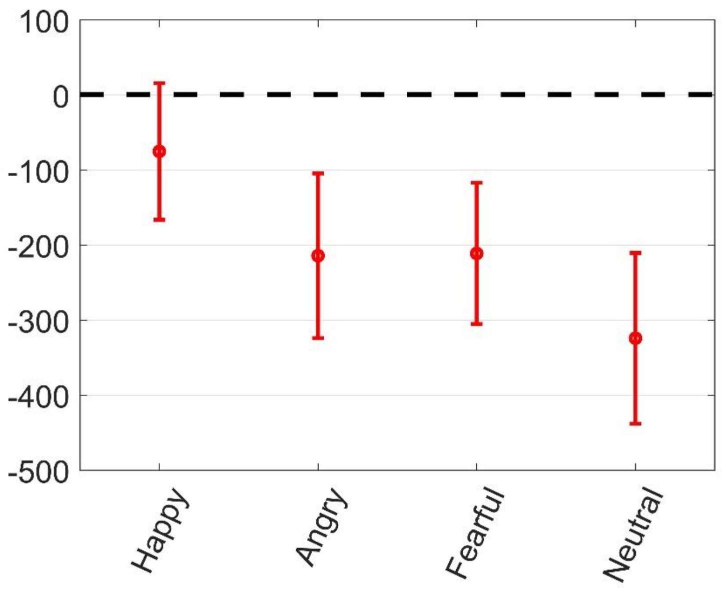

Figure S1. Effects of AOI (latency from mouth – latency from eyes) for the different emotions across groups. Error bars cover estimated marginal means covered by 95% confidence intervals.
